# Making Biodegradable Seedling Pots from Textile and Paper Waste—Part B: Development and Evaluation of Seedling Pots

**DOI:** 10.3390/ijerph18147609

**Published:** 2021-07-17

**Authors:** Jeanger P. Juanga-Labayen, Qiuyan Yuan

**Affiliations:** Environmental Engineering, Department of Civil Engineering, University of Manitoba, Winnipeg, MB R3T 5V6, Canada; jeanger.labayen@gmail.com

**Keywords:** biodegradable seedling pot, textile waste, paper waste, cotton, cardboard

## Abstract

This study evaluates the efficacy of using textile waste blended with paper waste to form biodegradable seedling pots. A bio-composite blend of cotton (20% cotton, 40% newspaper, and 40% corrugated cardboard) and polycotton (20% polycotton, 40% newspaper, and 40% corrugated cardboard) with an optimum strength was formed into seedling pots. The appreciated seedling pots (untreated blends of cotton and polycotton) were compared with the commercial pots (cardboard seed starter pot and Jiffy pot) in terms of mechanical properties (tensile strength and compressive strength), biodegradability (soil burial test and anaerobic digestion), and seed germination. The untreated blends of cotton and polycotton pots demonstrated a comparable optimum strength, while the Jiffy pot and cardboard seed starter pot obtained the least tensile and compressive strengths, respectively. The anaerobic biodegradability assay suggests that the cotton blend pot, polycotton blend pot, and cardboard seed starter pot can degrade anaerobically because of high biogas and methane generation potential. A 100% seed germination was observed from the four seedling pots tested. Thus, the results demonstrate the efficacy of utilizing textile waste and paper waste to develop seedling pots with desirable strength and biodegradability compared to the commercial pots.

## 1. Introduction

Staggering consumerism and economic growth have generated an unsustainable amount of discarded textile and paper waste in municipal solid waste (MSW) that ends up in landfills. The global expansion of the textile industry along with the consumers’ fast fashion trend makes clothing disposable, generating a massive amount of textile waste [1,2,3,4]. Textiles such as cotton and polycotton (60% cotton/40% polyester) are the predominant fibers that comprised most of the consumers merchandised [5,6,7]. Moreover, paper and paperboard waste represent the largest fraction of the total MSW (68.05 million tons) generated in the U.S. and the 3rd largest fraction of MSW disposed of in landfills [8]. Discarded textile and paper waste are fiber-rich resources that can be potentially used in the making of a biodegradable seedling pot. This study offers an environmentally sustainable option in diverting textile and paper waste from landfills by converting them into a biodegradable seedling pot.

Alternative containers or bio-containers were developed to promote sustainable greenhouse and nursery production that addressed the consumers’ “green” product perception and environmental sustainability. Bio-containers are made from biodegradable materials to provide alternative seedling pots replacing the non-renewable plastic containers. Bio-containers degrade naturally when planted or composted, which attracts sustainability and marketability [9]. Bio-containers can be classified into plantable and compostable based on their usage requirement and degradation rate [10,11]. Plantable bio-containers are planted directly in the ground can enhance the survival rate of plants by reducing root damage and transplanting fatigue as it eliminates the need to remove the pot before planting [12]. The factors that determine the pot biodegradation rate includes the nature of container material, soil quality (nutrients, moisture, pH, temperature, and microbial community), and climatic condition [9]. However, plantable bio-containers can rapidly decompose, yet they are durable enough for short-term production to withstand watering and handling requirements. Unlike plantable bio-containers, compostable bio-containers are not designed to be planted with the plant, instead, the pot should be removed from the plant before transferring into the final container or planting bed, and the used containers are composted separately [11]. Compostable bio-containers do not degrade quickly and the pot walls are strong enough to hinder the establishment of roots [13]. For this reason, containers must be removed before planting to be composted in a proper compost pile or composting facility to allow complete decomposition in a relatively short time [14].

Biodegradable pots are produced worldwide and are already being used in many greenhouses and nursery production facilities that promote sustainable organic gardening/farming. Some of the known biocontainers are CowPots (East Canaan, CT, USA) made of composted cow manure, compressed and held together with a binder; Jiffy pots (Jiffy Products, Kristiansand, Norway) are made from peat and paper fiber; and paper containers (Western Pulp Products, Corvallis, OR, USA, and Kord Products, Lugoff, SC, USA) are made from paper pulp with a binder [10]. Furthermore, the tensile strength of three selected biodegradable seedling plug-trays made from peat moss, wood fibre, and cow manure were in the range of 1.0–2.0 MPa [15]. Biodegradable pots made from recycled wastes of tomato (90%) and hemp fibers (10%) with sodium alginate as binder demonstrated a tensile strength of 1.20 MPa [16]. 

The development of bio-containers has been progressively focused on utilizing the appropriate biodegradable waste materials, improving the strength of the container, and increasing its biodegradability. Seedling pots made of recycled wastes of tomato and hemp fibres with sodium alginate as a binder enhance the development of roots and growth of plants [16]. Biosolids from wastewater treatment facility were utilized to develop a biosolids blend of cardboard and cellulose fibre with starch as a binder demonstrates an enhanced plant growth [17]. In a different study, pineapple waste was used to make decomposable pots of 1 cm thickness was decomposed after 45 days with a nitrogen and phosphorus release of 0.34% and 7.97 mg-P/kg, respectively [18]. Residues from sweet potato distillation with waste newspaper were potential to make recycled pots and observed that the plants’ roots penetrated through the pot without causing damage to the plant and the pot served as a fertilizer to the plant upon decomposition [19]. Seedling pots made from biomaterials and banana peels suggested that the higher content of banana peels (70%) could accelerate the biodegradability of the pot [20].

Thus far, in terms of waste material utilization, no available studies have been conducted on developing biodegradable seedling pots using textile waste (cotton and polycotton) blended with paper waste (newspaper and corrugated cardboard). The durability of bio-container is one of the factors considered by the nursery and greenhouse industry prior to utilization [21]. Tensile strength accounts for the handling capacity of biodegradable seedling pots [22]. Typically, tensile forces are exerted on the container walls during plant growth and manually transporting the container [17]. This study determines an optimum bio-composite blend of cotton and polycotton in terms of tensile strength and bending strength tests. The resulting optimum bio-composite blends of cotton and polycotton were used to develop a bio-composite seedling pot. Furthermore, these formulated seedling pots were compared with the commercially available seedling pots (cardboard seed starter pot and Jiffy pot) in terms of tensile and compressive strengths. A compressive strength test was performed on the seedling pots to determine the pot capacity to withstand compression load that can be exerted by the seedling roots along the walls of the container during plant growth.

## 2. Materials and Methods

### 2.1. Preparation of Bio-Composite Sheets

Blending of paper substrates (newspaper and corrugated cardboard) with cotton and polycotton to form bio-composite sheets and determining the tensile and bending strengths are worthy to be investigated prior to making a biodegradable seedling pot [23]. The substrates used in this study include textile waste in the form of soiled towel (100% cotton), polycotton fabric (60% cotton/40% polyester), and paper waste in the form of used newspaper and corrugated cardboard. The polycotton fabric has a polyester component, which is synthetic and non-biodegradable. Three different blends of cotton and polycotton with paper waste were considered to form bio-composite sheets (Table 1). Except for corrugated cardboard (soaked in deionized water), the substrates were soaked in 5% NaOH for 5 h and rinsed using deionized water. Thereafter, the substrates were blended into pulp using a blender (Pro-vita, electric power blender, 1400 W (Thinkkitchen, Windsor, ON, Canada) and a freshly prepared binder (20% cornstarch) was added into a drained blended pulp and weighed accordingly so that each sheet prepared for tensile strength and bending strength tests should have 0.5 g TS and 1.3 g TS, respectively.

**Figure 1:**
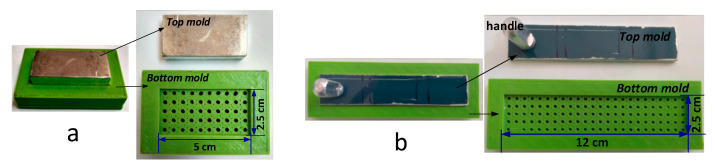
Molds for bio-composite sheet for testing the (**a**) tensile strength and (**b**) bending strength.

The bio-composite sheet was formed using 5 cm × 2.5 cm and 12 cm × 2.5 cm molds for tensile strength and bending strength tests, respectively (Figure 1). The compression method and instrumentation used to form sheets were common with Part A of this study [23]. After the compression, the bio-composite sheet was removed from the mold and dried for 5 h at 105 °C and kept in the desiccator prior to testing. Six sheets for each blend were prepared and tested for tensile and bending strengths to determine an optimum bio-composite blend.

The addition of binder remarkably improved the tensile strength by 180–395%, while the alkali treatment (5% NaOH for 5 h soaking) increased the tensile strength by 14–21% only [23]. From these results, untreated bio-composite sheets for C blend 1 and PC blend 1 were considered as control treatments to compare the tensile and bending strengths with the treated bio-composite sheets. 

### 2.2. Preparation of Bio-Composite Pots

The bio-composite pots were prepared after evaluating the results of the previous tests. Untreated bio-composite blends (C blend 1 and PC blend 1) were prepared to form seedling pots. The formulated seedling pots from this study were compared with the commercially available biodegradable seedling pots, the Jiffy-Pots 2 and cardboard seed starter pot, bought from Dollarama store, Winnipeg, Canada. The Jiffy pot (size: 2 in diameter) is made of Canadian Sphagnum peat moss and wood pulp by Jiffy Group, Canada. The cardboard seed starter pot (size: 2.5 in diameter) is made of cardboard by Seeders, China. The average dry mass of the commercial pots (cardboard seed starter pot and Jiffy pot) was determined in this study and found to be 4 g TS per seedling pot and this was used as a basis to prepare the bio-composite pots. 

**Figure 2:**
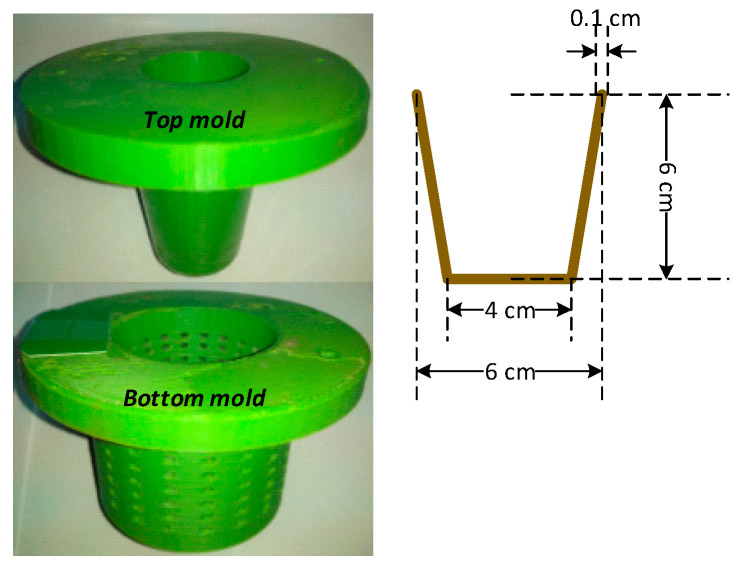
Mold used to form seedling pot.

The seedling pot was formed using a mold as illustrated in Figure 2. The mold was drafted using Solid Works software and created by a 3D printer machine using acrylonitrile butadiene styrene (ABS) plastic material. The weighed substrate for each blend of cotton and polycotton was soaked in deionized water for 5 h prior to pulping. The resulting mixture after binder addition was manually placed to cover the entire bottom mold. Following that, the top mold was placed atop the bottom mold for compression using a load of 500 N. Six seedling pots for each blend were prepared and tested for compressive strength. 

### 2.3. Mechanical Tests

#### 2.3.1. For Bio-Composite Sheet

**Figure 3:**
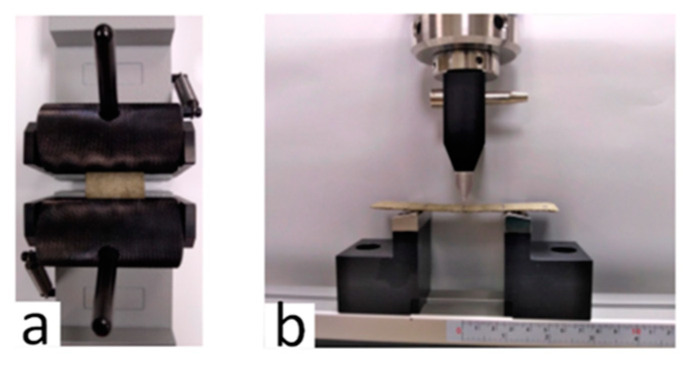
Mechanical tests performed on bio-composite sheets (**a**) tensile strength and (**b**) bending strength.

A universal testing machine (LS5 Model, Lloyd Materials Testing, Lloyd Instrument Ltd., West Sussex, UK) equipped with 5 kN load cell was used to determine the tensile strength and bending strength of the prepared bio-composite sheets (Figure 3). The method used to perform tensile strength was common with Part A of this study [23]. For bending strength, a three-point bending test was used. The sheet with an average dimension of 110 mm × 23.5 mm × 1.20 mm was placed on the bending beam at a span length of 50 mm. The test was performed at an extension rate of 2 mm/min and was set to stop when the sample breaks. 

#### 2.3.2. For Seedling Pots

**Figure 4:**
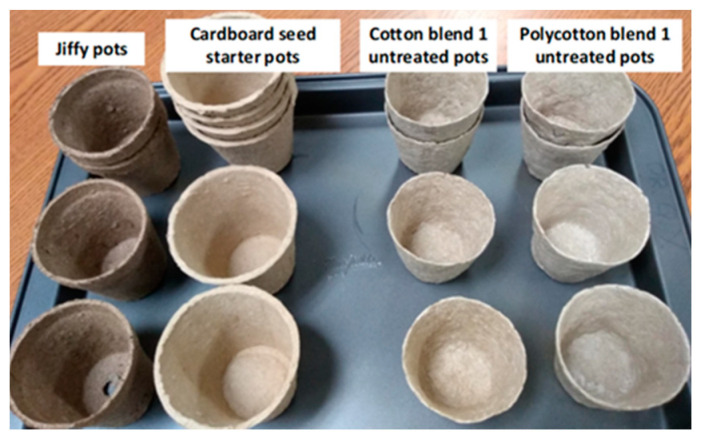
Seedling pots used for compressive strength test.

Tensile strength and compressive strength tests were performed on the formulated seedling pots and the commercial pots (cardboard seed starter pot and Jiffy pot). For tensile strength, sheets with dimensions 5 cm × 2.5 cm were cut from the walls of the Jiffy pot and cardboard seed starter pot. Figure 4 shows the seedling pots that were subjected to compressive strength test. 

### 2.4. Degradability Test

#### 2.4.1. Anaerobic Degradability Test

The anaerobic degradability of optimized bio-composite pots (the untreated cotton blend and polycotton blend) along with the two commercial pots (cardboard seed starter pot and Jiffy pot) were investigated. This test determines biodegradability as a measure of specific methane yield and biogas yield, % chemical oxygen demand (COD) reduction, and % volatile solids (VS) reduction. The seedling pots were cut into smaller pieces and further size reduction was performed using a kitchen coffee grinder. As shredding or grinding as part of substrate particle size reduction is recommended prior to performing for anaerobic degradability test [24,25]. The substrates were weighed and inoculum was added to attain the substrate to inoculum ratio (SIR) of 0.5. SIR is one of the important parameters that affects the process stability of anaerobic digestion and the VS concentration of inoculum should be always higher compared to that of the substrates [26]. Furthermore, the recommended SIR should be above 0.1 [27]. The effect of different SIR of 0.5, 1, 1.5, and 2 using cotton substrate revealed that the SIR of 0.5 has the highest biogas and methane yields and increasing the SIR over 0.5 can negatively affect the anaerobic digestion performance [28]. Blank reactors containing inoculum and deionized water with a resulting concentration of 5 g VS/500 mL were included as control to determine the biogas and methane yields from the inoculum. The background methane produced from the blank assays is subtracted from the methane generated from the substrate [25]. 

**Figure 5:**
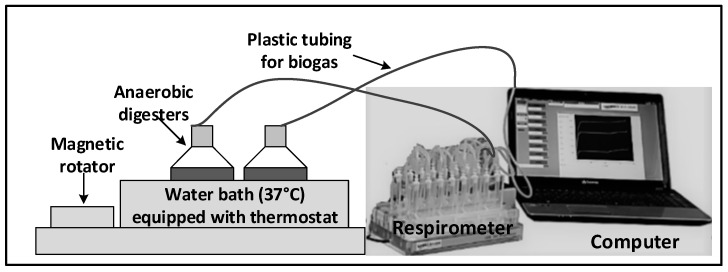
Anaerobic degradability assay.

A mesophilic anaerobic inoculum in the form of digested sludge was obtained on 25 June 2019 from the North End Water Pollution Control Centre (NEWPCC), Winnipeg, Manitoba, Canada with an estimated solid retention time (SRT) of 12–15 days. The sludge was pre-incubated at 37 °C for two days to be degassed [25]. The inoculum was transferred into the anaerobic vessels via plastic tubing to reach the bottom part of the vessel whilst minimizing air entrapment to maintain the anaerobic condition. The physico-chemical characteristics of the degassed inoculum includes TS of 18,906.33 mg/L, VS of 10,999.33 mg/L, COD of 16,402.62 mg/L and pH of 7.69. Figure 5 depicts the anaerobic digestion set-up using research respirometer equipment (AER 800, Challenge Technology, Springdale, AR, USA) [28]. Fifteen Wheaton laboratory glass bottles (Sigma-Aldrich, Oakville, ON, Canada) with a total volume of 775 mL and a working volume of 500 mL were used as digesters. The digesters were filled with inoculum and substrate at SIR of 0.5 in triplicates. To determine the endogenous gas production for the mesophilic anaerobic inoculum, blank bottles were prepared in triplicates without substrate addition but with the same amount of anaerobic inoculum. The bottles were sealed and flushed with 100% nitrogen gas to establish an anaerobic condition. The digesters were continuously monitored to produce biogas until they reached a plateau after the 20-day period.

#### 2.4.2. Soil Burial Test

**Figure 6:**
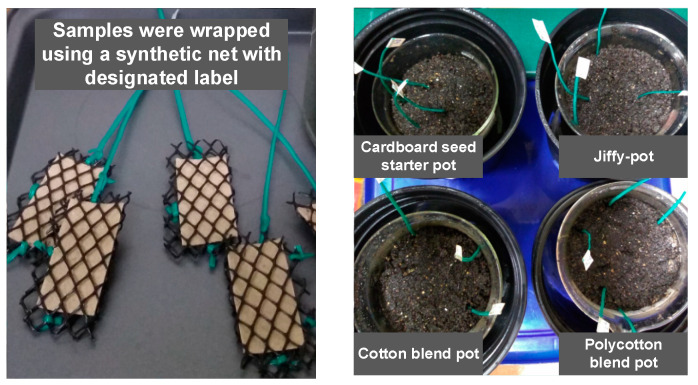
Soil burial test using different seedling pots.

The method used for soil burial study was adapted from SR EN ISO 846/2000 [29,30]. This study used a fine-grained natural active soil consisting of equal parts of garden soil and compost from yard waste of about 1 kg. The soil was passed through a screen with a mesh size of 2 mm. The soil water content was adjusted to 60 ± 5% by using an aqueous solution of 1 g NH_4_NO_3_ and 0.2 g K_2_HPO_4_ per liter of water. The mean pH of soil (20 g soil in 20 mL deionized water) was 7.30, which is within the pH range (6 to 8) set for soil [31]. Samples from the side walls of the seedling pots were cut into 5 cm × 2.5 cm sheets and weighed using an analytical scale with a precision of 0.0001 g. Four replicates of sheets for each seedling pot were buried vertically in soil (Figure 6). The samples were incubated at 25 °C for a total of 4 months. At the end of each testing interval (15 days, 30 days, 45 days, 60 days, 75 days, 90 days, 105 days, and 120 days), the sheet samples were removed from the soil, washed with deionized water to remove adherent soil, and dried on paper wipes and stored in the desiccator for at least 48 h until a constant weight can be attained. Sheet samples were weighed at the beginning and at the end of each test interval and the weight loss signifies the degree of degradation. The total % weight loss after 4 months was determined (Equations (1) and (2)).

(1) $$\%\;\mathrm{wt}.\;\mathrm{loss}=\frac{\mathrm{Initial}\;\mathrm{wt}.{\;\mathrm{at}\;\mathrm{day}}_{0}-\;\mathrm{Final}\;\mathrm{wt}.{\;\mathrm{after}\;\mathrm{day}}_{15}}{\mathrm{Initial}\;\mathrm{wt}.{\;\mathrm{at}\;\mathrm{day}}_{0}}\times 100\;$$

(2) $$\%\;\mathrm{wt}.{\;\mathrm{loss}}_{\left(\mathrm{after}\;\mathrm{every}\;15\;\mathrm{days}\right)}=\frac{\mathrm{Initial}\;\mathrm{wt}.{\;\mathrm{before}\;\mathrm{day}}_{15}-\;\mathrm{Final}\;\mathrm{wt}.{\;\mathrm{after}\;\mathrm{day}}_{15}}{\mathrm{Initial}\;\mathrm{wt}.{\;\mathrm{before}\;\mathrm{day}}_{15}}\times 100\;$$

### 2.5. Germination Test

A seed germination assay was prepared using a saturated aqueous extract of untreated cotton blend pot, untreated polycotton blend pot, cardboard seed starter pot, and Jiffy pot. The dried pots were cut into smaller pieces, ground using a coffee grinder, weighed, and transferred into a flask. The amount of deionized water added into the flask was 5 times the weight of the pot (1:5). The flasks were covered and placed in a mechanical shaker for 1 h. Thereafter, the suspension was filtered using a Buchner filtration setup [32]. Germination testing was performed to determine the toxicity of the studied seedling pots on four different seeds (lettuce, navy bean, soybean, and mung bean). Tests were performed by placing two discs of Whatman qualitative filter paper in a disposable plastic petri dish (100 mm × 15 mm). Ten identical undamaged seeds for each seed variety were spaced out evenly on the filter paper in each dish, and were subsequently wetted with 4 mL of the saturated extract aqueous solution [33]. Triplicates were performed for each seed using four different extract solutions. For each seed variety, a triplicate control dish using deionized water was also included. All dishes were incubated at 25 °C for 7 days under dark conditions. After 7 days, the seed germination (SG) and the relative seed germination (RSG) were determined (Equations (3) and (4)) [34]. The seed was considered to be germinated when the length of the primary root grew a minimum of 5 mm [33].

(3) $$\mathrm{SG}=\frac{\mathrm{Number}\;\mathrm{of}\;\mathrm{germinated}\;\mathrm{seeds}}{\mathrm{Number}\;\mathrm{of}\;\mathrm{total}\;\mathrm{seeds}}\times 100\%\;$$

(4) $$\mathrm{RSG}=\frac{\mathrm{Number}\;\mathrm{of}\;\mathrm{germinated}\;\mathrm{seeds}\;\left(\mathrm{sample}\right)}{\mathrm{Number}\;\mathrm{of}\;\mathrm{germinated}\;\mathrm{seeds}\;\left(\mathrm{control}\right)}\times 100\%$$

## 3. Results and Discussion

### 3.1. Tensile Strength and Bending Strength of Bio-Composite Sheet

**Figure 7:**
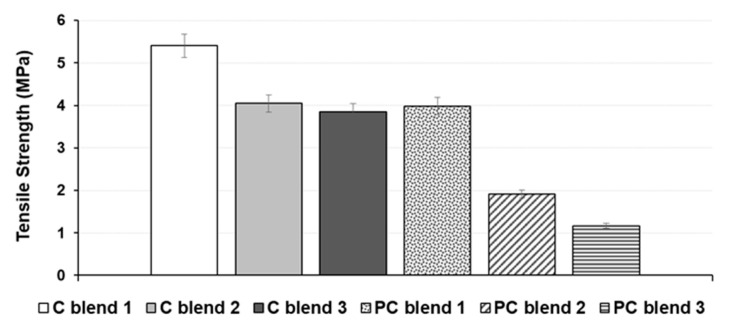
Tensile strength of bio-composite blends of cotton and polycotton.

**Figure 8:**
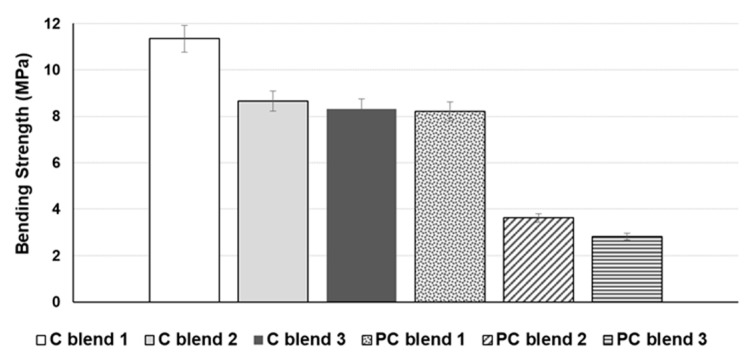
Bending strength of bio-composite blends of cotton and polycotton.

The C blend 1 bio-composite sheet achieved optimum tensile and bending strengths of 5.40 MPa and 11.34 MPa, respectively, amongst all the cotton blends (Figure 7 and Figure 8). Furthermore, for polycotton blends, the same trend was observed; the PC blend 1 achieved an optimum tensile and bending strengths of 3.99 MPa and 8.22 MPa, respectively, compared to the other blends. Thus, C blend 1 and PC blend 1 were chosen as the optimum blends that achieved the highest tensile and bending strengths. It should be noted that the C blend 1 consists of 20% cotton, while the PC blend 1 contains 20% polycotton (60% cotton, 40% polyester). The higher cellulose content of cotton blend 1 can be attributed to its higher tensile and bending strengths. Cellulose has a linear framework of semicrystalline structure that provides fiber strength, stiffness, and stability [35,36]. Additionally, the use of cornstarch as a natural binder provides good adhesion mechanism with C blend than the PC blend bio-composite sheets. Furthermore, the identical nature of binder matrix and pulp fibres which is made of cellulose are fully compatible to allow efficient stress transfer and adhesion [37,38]. To date, studies on the mechanical properties of biodegradable seedling pots are considered limited. A bio-composite sheet made of recycled wastes of tomato, hemp fibres, and sodium alginate binder attained an optimum tensile strength of 1.2 MPa [16]. 

### 3.2. Tensile Strength and Bending Strength of Treated and Untreated Bio-Composite Sheets 

**Figure 9:**
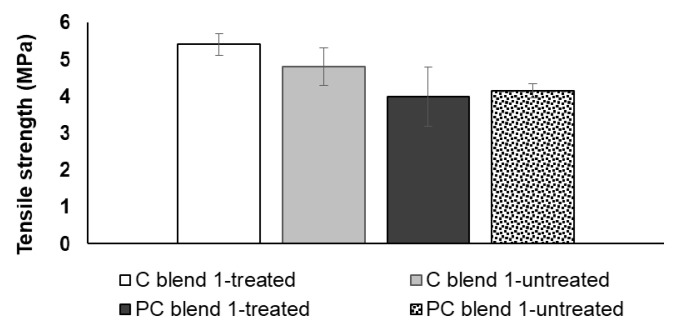
Tensile strength of treated and untreated cotton and polycotton blends.

**Figure 10:**
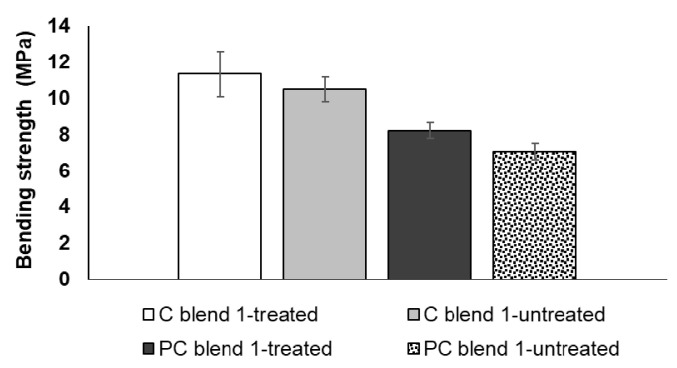
Bending strength of treated and untreated cotton and polycotton blends.

Comparable tensile strength was observed for treated and untreated blends of cotton and polycotton sheets (Figure 9). Additionally, commensurate bending strength results were observed for treated and untreated blends of cotton and polycotton sheets (Figure 10). The results suggest that alkali treatment has no beneficial effect on enhancing the strength of bio-composite sheets. This further signifies that treatment is non-essential in the preparation of the seedling pots. The exclusion of alkali treatment in the process provided added benefits of water conservation as rinsing is no longer necessary and thereby minimizing wastewater.

### 3.3. Tensile Strength and Compressive Strength of Seedling Pots

**Figure 11:**
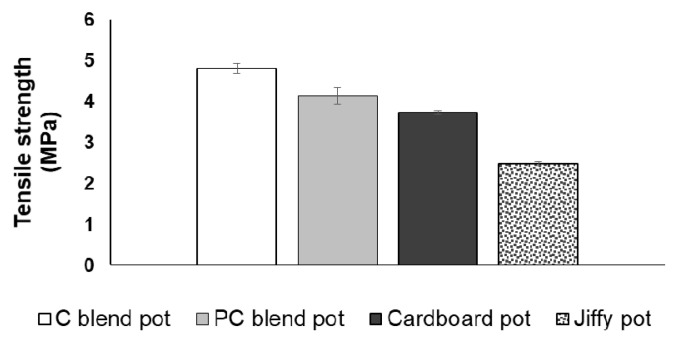
Tensile strength of the developed seedling pots and the commercial pots.

**Figure 12:**
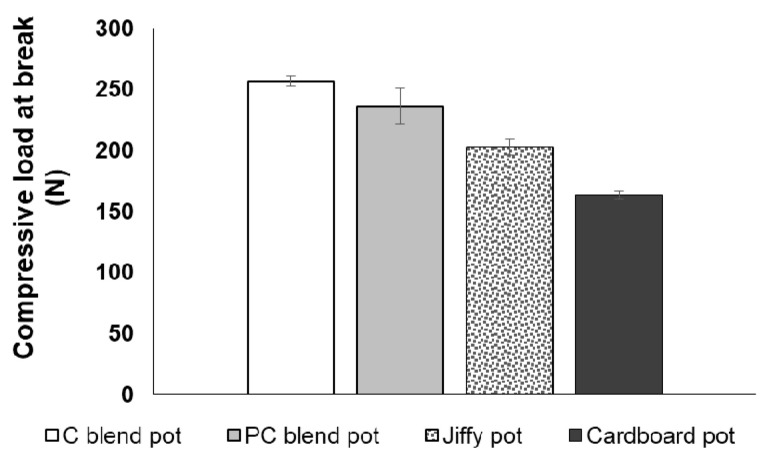
Compressive strength of the developed seedling pots and the commercial pots.

The formulated seedling pots attained higher tensile and compressive strengths as compared to the commercial pots (Figure 11 and Figure 12). Specifically, the cotton blend pot achieved the optimum tensile and compressive strengths of 4.81 MPa and 256.64 N, respectively. The higher cellulose content of the cotton blend pot and the use of cornstarch as a natural binder provides a good adhesion mechanism between the binder matrix and the pulp fibres, which allow efficient stress transfer and adhesion [37,38]. For the commercial seedling pots, the cardboard seed starter pot has a higher tensile strength (3.74 MPa) as compared to the Jiffy pot. On the other hand, the Jiffy pot obtained higher compressive strength (202.86 N) as compared to the cardboard seed starter pot. The above results clearly substantiate higher strength characteristics for the cotton blended and polycotton blended pots as compared to the commercially available pots. Thus far, published articles on the mechanical properties of biodegradable seedling pots are considered limited. The tensile strength of three selected biodegradable seedling plug-trays made from peat moss, wood fibre, and cow manure was in the range of 1.0–2.0 MPa [15], which is lower compared to the optimum tensile strength found in this study. 

### 3.4. Anaerobic Biodegradability of Seedling Pots

**Figure 13:**
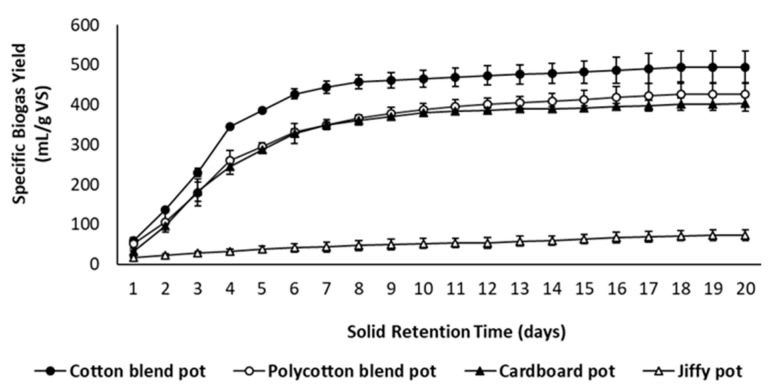
Specific biogas yield of seedling pots.

**Figure 14:**
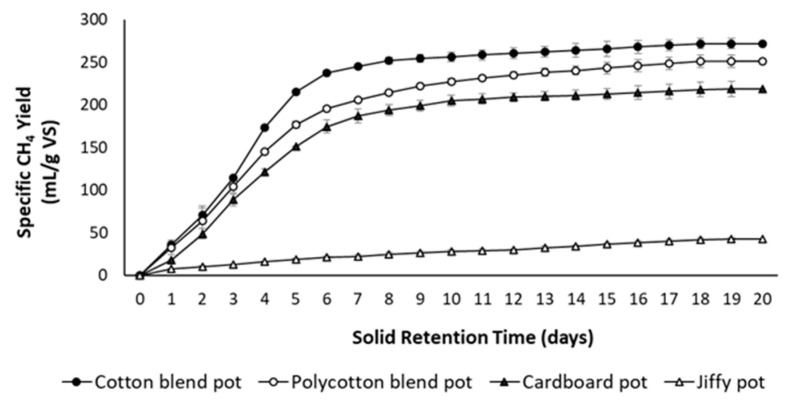
Specific methane yield of seedling pots.

**Figure 15:**
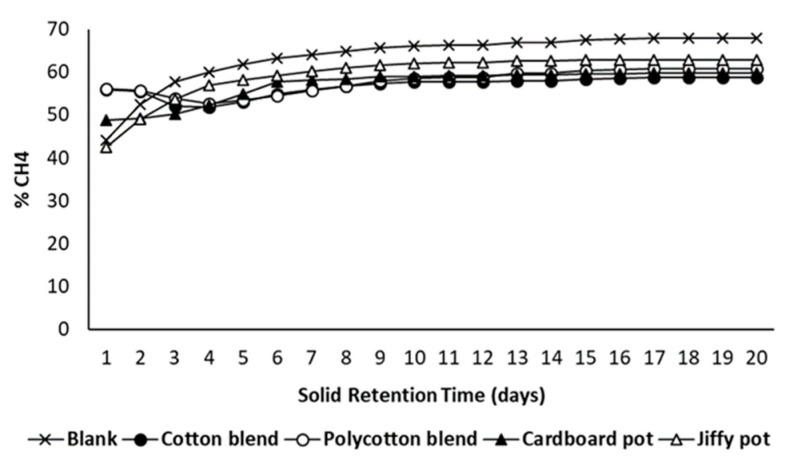
Methane concentration of seedling pots.

Figure 13 and Figure 14 present the specific biogas and methane yields for the studied seedling pots, respectively. The cotton blend pot produced the highest biogas (494.30 mL/g VS) and methane yields (271.80 mL/g VS) as compared to the polycotton blend pot because the polycotton blend pot contains polyester fraction, which is synthetic or non-biodegradable. Unlike polycotton, cotton consists of higher cellulose content, which is a potential substrate for biological conversion [39]. Additionally, the formulated pots (cotton and polycotton blend) produced higher biogas and methane yield as compared to the commercial pots (cardboard seed starter pot and Jiffy pot). Moreover, the cardboard seed starter pot generated an appreciable biogas and methane yield as compared to the Jiffy pot. The methane concentration rose above 50% after 4 days of anaerobic digestion for all the reactors indicating an active methane phase (Figure 15). Furthermore, a shorter digestion period of 10 days is possible to yield optimum biogas and methane production, which is marked by the presence of a sharp plateau. The solids analysis of the studied seedling pots is presented in Table 2.

An active methane phase is indicated the production of at least 50% CH_4_ concentration in biogas and a pH between 7 and 8 [40,41,42]. After 20 days of anaerobic digestion, the measured pH values were 7.52, 7.16, 7.23, 7.16 and 7.22 for blank, cotton blend pot, polycotton blend pot, cardboard seed starter pot, and Jiffy pot, respectively. The pH values above 7 indicate a stable methane phase for all the pots. However, the digester that contains the Jiffy pot substrate shows a stable pH condition and methane concentration above 50% and restricted anaerobic degradation was observed because of its lower biogas and methane potential. This can be further explained by the % VS reduction and % COD reduction. Among the seedling pots studied, the Jiffy pot attained the lowest % VS reduction and % COD reduction of 32% and 20%, respectively. This indicates that the Jiffy pot has a lower potential to degrade anaerobically. Interestingly, the % VS reduction for cotton blend pot, polycotton blend pot and cardboard pot was 56%, 51% and 50%, respectively, which complements with the % COD reduction of 66% for the cotton blend pot, polycotton blend pot, and cardboard seed starter pot.

### 3.5. Soil Burial Test 

**Figure 16:**
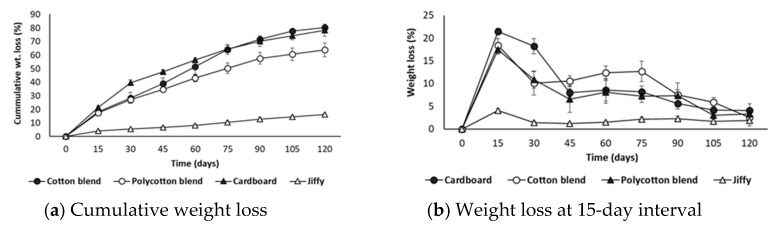
Soil burial test results.

The cumulative percent weight loss of the samples buried in soil determines the degree of degradation. The highest percent weight loss of 80% and 78% was obtained for cotton blend and cardboard seed starter pot, respectively after 120 days (Figure 16a). A lower weight loss of 64% from the polycotton blend can be attributed to its polyester content, which is synthetic and non-biodegradable. The desirable weight loss observed for cotton and polycotton blends can be attributed to the presence of cornstarch as a binder; as the addition of starch increased the film weight loss in soil burial test [43]. The Jiffy pot attained the lowest degradability of 16%. The French specification (NF U52-001) defines the criteria for soil biodegradability as a minimum of 60% and a maximum of 90% if soil (for 12-month period) and compost media (for 6 months) are used [31]. This denotes that the cotton and polycotton blend pots and the cardboard seed starter pot meet the French standard for soil biodegradation criteria. Figure 16b presents the percent weight loss at every 15-day interval and shows a significant percent weight loss after the first 15 days for all the samples. Almost 50% of the weight loss was achieved after 45 days; however, significant weight loss extends until 75 days for the cotton blend. The results from the anaerobic degradability test are also compatible with the soil burial tests.

### 3.6. Germination Test

**Figure 17:**
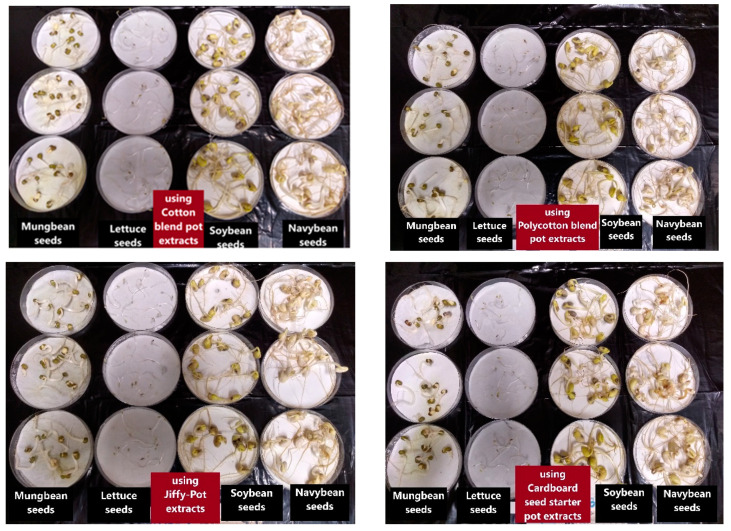
Germination tests of four seeds using cotton blend pot aqueous extract.

The pH and conductivity values of the aqueous extracts are given in Table 3. The pH of an aqueous solution extracted from Jiffy pot was lower than the other studied pots, this is because the Jiffy pot used in this study is made of Sphagnum peat moss and wood pulp. The pH result observed commensurate with the pH range of peat soil of 3.7–5.2 with an average pH of 4.5 [44]. After 7 days of incubating the dishes with seeds, the SG and the RSG for all the seeds tested from the four saturated aqueous pot extracts were 100%. Figure 17 represents a photograph of the germinated seeds using aqueous extract from the cotton blend pot. This shows that all the seedling pots do not have a toxicity effect on seed germination. However, to gain a better understanding of the germination test, it is worthwhile to determine the daily germination rate in terms of the daily growth of seed using different aqueous pot extracts. 

## 4. Conclusions

This study evaluated the efficacy of utilizing textile waste (cotton and polycotton) blended with paper waste (newspaper and corrugated cardboard) to develop biodegradable seedling pots. The cotton blend pot and polycotton blend pot achieved higher tensile strength and compressive strength as compared to the commercial seedling pots. The anaerobic biodegradability assay suggests that the cotton blend pot, polycotton blend pot, and cardboard seed starter pot can degrade anaerobically because of their higher biogas and methane generation potential. After a 120-day soil burial test, the degree of degradation for cotton blend pot, cardboard seed starter pot, and polycotton blend pot was higher than the Jiffy pot, which further ascertains the utilization of the formulated pots. A 100% seed germination from the four seedling pots using lettuce, navy bean, soybean, and mung bean seeds implies that the seedling pots have no toxicity effect on the seed growth. Thus, the results demonstrated that the developed seedling pots obtained an optimum strength and biodegradability compared to the tested commercial seedling pots. Further research towards the use of local organic waste to be blended with textile waste to make biodegradable seedling pots. Nonetheless, it could be worthwhile comparing the daily seed growth or germination rate using different aqueous pot extracts. 

## Figures and Tables

**Table 1:** Composition of bio-composite sheets.

Bio-Composite Sheets	Substrate Composition		
Cotton (C) blend	Cotton	Newspaper	Corrugated cardboard
C blend 1	20%	40%	40%
C blend 2	50%	25%	25%
C blend 3	80%	10%	10%
Polycotton (PC) blend	Polycotton	Newspaper	Corrugated cardboard
PC blend 1	20%	40%	40%
PC blend 2	50%	25%	25%
PC blend 3	80%	10%	10%

**Table 2:** Solids analysis of seedling pots used for anaerobic digestion.

Substrates	Moisture Content (%)	Total Solids (%)	Volatile Solids (%)
Cotton blend pot	4.11	95.89	95.60
Polycotton blend pot	4.20	95.80	95.80
Cardboard seed starter pot	3.53	96.47	83.94
Jiffy pot	6.18	93.82	96.90

**Table 3:** pH and conductivity of aqueous extracts from studied pots.

Aqueous Extracts	pH	Conductivity (µS/cm)
Cotton blend pot	7.37	206.30
Polycotton blend pot	7.40	220.20
Cardboard pot	7.39	614.10
Jiffy pot	4.72	236.60
